# Serial Ultrasound-Guided Carotid Artery Compression During Chest Compressions for Assessing Circulatory Status: A Prospective Pilot Diagnostic Study

**DOI:** 10.3390/diagnostics16182956

**Published:** 2026-09-13

**Authors:** Seung Jin Maeng, Hee Yoon, Ik Joon Jo, Sejin Heo, Hansol Chang, Gun Tak Lee, Jong Eun Park, Taerim Kim, Se Uk Lee, Sung Yeon Hwang, Jihyeon Kim, Min Ji Kim

**Affiliations:** 1 Department of Emergency Medicine, Samsung Medical Center, Sungkyunkwan University School of Medicine, Seoul 06351, Republic of Korea; 2 Department of Emergency Medicine, Graduate School, College of Medicine, Kyung Hee University, Seoul 02447, Republic of Korea; 3 Department of Emergency Medicine, Denver Health, Denver, CO 80204, USA; 4 Research Institute for Future Medicine, Samsung Medical Center, Seoul 06351, Republic of Korea; 5 Biomedical Statistics Center, Research Institute for Future Medicine, Samsung Medical Center, Seoul 06351, Republic of Korea

**Keywords:** cardiopulmonary resuscitation, ultrasonography, carotid arteries, return of spontaneous circulation, pulse, point-of-care systems

## Abstract

**Background** Point-of-care ultrasound-carotid artery compression (POCUS-CAC) is a technique using ultrasound to assess carotid artery compressibility during pulse checks. We expanded the use of POCUS-CAC during ongoing chest compressions for identifying circulatory status at the subsequent rhythm check. **Methods** A prospective, single-center study was conducted on patients aged ≥18 years presenting to the emergency department with cardiac arrest between June 2022 and October 2023. POCUS-CAC was performed at approximately 30 s intervals during ongoing chest compressions whenever clinically feasible. A POCUS-CAC assessment was classified as ROSC-compatible if the carotid artery remained non-compressible during both the systolic and diastolic phases of chest compressions. Circulatory status at the subsequent rhythm check was determined according to the 2020 American Heart Association guidelines. The diagnostic performance was evaluated using conventional diagnostic performance measures, with 95% confidence intervals estimated using patient-level cluster bootstrap resampling. **Results** Among 37 patients, 296 POCUS-CAC assessments were analyzed. POCUS-CAC during ongoing chest compressions demonstrated 90.6% sensitivity, 84.9% specificity, and 87.2% accuracy for identifying circulatory status at the subsequent rhythm check. In exploratory longitudinal analyses, diagnostic performance remained generally consistent across the first three assessments within cardiopulmonary resuscitation cycles. **Conclusions** POCUS-CAC during ongoing chest compressions showed favorable diagnostic performance for identifying circulatory status at the subsequent rhythm check. Serial POCUS-CAC assessments may provide additional information regarding changes in circulatory status during ongoing CPR. Further prospective multicenter studies are needed to validate these findings.

## 1. Introduction

The timely identification of return of spontaneous circulation (ROSC) during cardiopulmonary resuscitation (CPR) is critical for determining the appropriate resuscitation strategy [1,2]. ROSC signals the restoration of effective cardiac function and blood flow. However, recognizing ROSC amidst ongoing chest compressions poses a significant challenge owing to the difficulty in differentiating between the effects of chest compressions and actual spontaneous circulation. Current methods for detecting ROSC, such as manual palpation of central pulses combined with an organized rhythm, require intermittent pauses in chest compressions [3]. Although these interruptions are necessary, they can negatively impact the efficacy of CPR by reducing coronary and cerebral perfusion [1,4]. Consequently, there is a growing interest in finding non-invasive, continuous monitoring techniques that can identify ROSC without interrupting chest compressions.

Point-of-care ultrasound (POCUS) has emerged as a valuable tool in the management of cardiac arrest, allowing for real-time visualization of cardiac activity and identification of reversible causes [5]. POCUS-carotid artery compression (POCUS-CAC) is a novel pulse check technique, which involves using an ultrasound probe to apply pressure on the carotid artery while simultaneously assessing the compressibility and pulsatility of the artery [6,7,8]. This method reduces the median time for pulse identification to around two seconds, markedly enhancing ROSC detection speed compared to manual pulse checks [9,10,11]. Furthermore, Chang et al. observed a case in which ROSC was predicted during ongoing chest compression when the carotid artery did not collapse despite probe compression [12]. Similarly, Kang et al. predicted ROSC in six out of ten cases during chest compression using POCUS-CAC, in which the carotid artery did not collapse under probe compression while the vein still did, confirming ROSC at the subsequent pulse check [9]. However, evidence regarding the diagnostic performance of POCUS-CAC during ongoing chest compressions remains limited.

We aimed to investigate the feasibility and diagnostic performance of POCUS-CAC during ongoing chest compressions for identifying circulatory status at the subsequent rhythm check. We also explored the serial behavior of POCUS-CAC assessments during ongoing CPR.

## 2. Patients and Methods

### 2.1. Study Design and Setting

A single-center prospective study was conducted in an adult emergency department (ED) of a tertiary academic medical center in South Korea, which manages an average of 200 patients per day. The study period was from June 2022 to October 2023. This study was approved by the Institutional Review Board of our institution, and the requirement of informed consent was waived by the review board (IRB file number 2022-04-016). Patients were unable to be involved in any part of the research beyond being study subjects due to their cardiac arrest status. All procedures were performed in accordance with the principles of the Declaration of Helsinki.

### 2.2. Study Population

This study included patients aged ≥18 years who presented to our ED with either out-of-hospital cardiac arrest (OHCA) or in-hospital cardiac arrest. The major exclusion criteria were head or neck trauma or abnormal neck anatomy due to prior surgical or oncological treatments that disturbed the application of ultrasound on the neck. Patients in whom the application of POCUS-CAC was not feasible during critical CPR situations, including cases of manpower shortages or insufficient CPR duration, were also excluded.

### 2.3. Study Protocol

**Figure 1:**
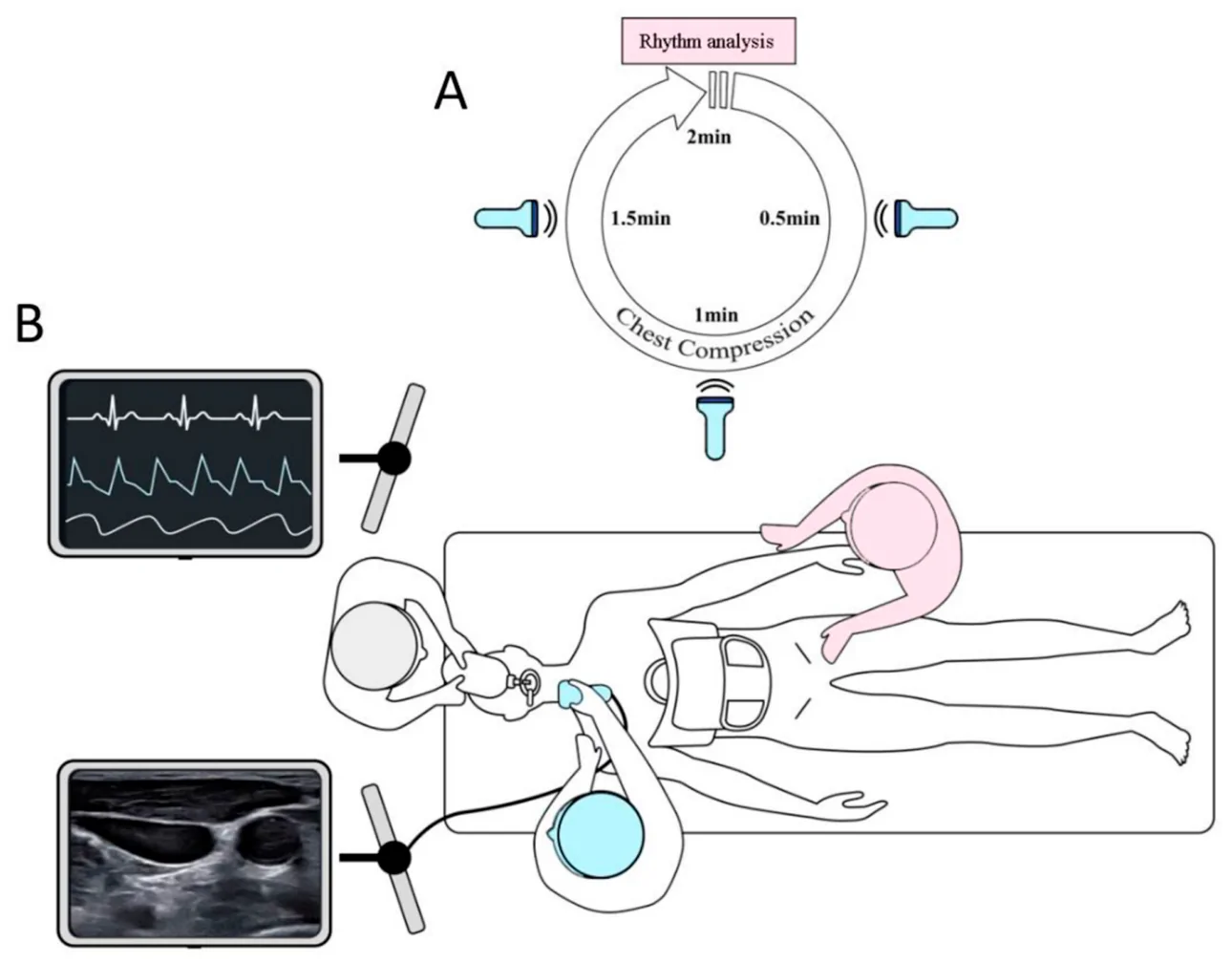
Study protocol. (**A**) Illustration of the intended POCUS-CAC evaluation cycle during CPR at approximately 30 s intervals. (**B**) Basic layout of the healthcare providers involved in this study, presenting their roles during resuscitation efforts. Blue: emergency physician who checks POCUS-CAC; pink: a clinician who checks manual pulsation every 2 min. Abbreviations. CPR, cardiopulmonary resuscitation; POCUS-CAC, point-of-care ultrasound-carotid artery compression.

In line with the 2020 Advanced Cardiovascular Life Support (ACLS) guidelines [3], high-quality CPR was immediately initiated for patients experiencing cardiac arrest, and carotid ultrasound was performed. POCUS-CAC was applied at approximately 30 s intervals during ongoing chest compression whenever clinically feasible to evaluate carotid artery compressibility based on ultrasound findings. At the subsequent scheduled rhythm check within each 2 min CPR cycle, another experienced clinician, blinded to the ultrasound results, determined the circulatory status according to ACLS guidelines, based on manual pulse palpation and the patient’s electrocardiogram (ECG) rhythm (Figure 1). Each POCUS-CAC assessment obtained within a CPR cycle was paired with the circulatory status determined at the subsequent rhythm check; therefore, multiple POCUS-CAC assessments within the same cycle were assigned to the same reference-standard outcome. The chronological sequence of POCUS-CAC assessments within each cycle was preserved; however, the nominal 30 s intervals recorded in the CRF represented the intended acquisition schedule rather than exact acquisition timestamps.

Following the established POCUS-CAC protocols from previous studies, a linear probe was placed transversely across the middle one-third portion of the patient’s neck to identify both the carotid artery and the internal jugular vein [9]. During ongoing chest compressions, the examiner applied pressure to the probe perpendicular to the skin for up to 2 s at approximately 30 s intervals whenever clinically feasible. To standardize the degree of externally applied probe pressure, complete collapse of the internal jugular vein was used as an anatomical reference during compression. Once complete internal jugular vein collapse was achieved, no additional probe pressure was applied. Depending on the clinical situation, either the right or left carotid artery was used. Subsequently, the examiner visually assessed the carotid artery’s compressibility during both the systolic phase, influenced by the compressions, and the diastolic phase, occurring during chest recoil. A ROSC-compatible status was recorded if the carotid artery was non-compressible in both phases (Supplementary Video S1A). An arrest-compatible finding was recorded if the carotid artery appeared compressed during the diastole period (Supplementary Video S1B). The ultrasound examiners did not share the results of POCUS-CAC with CPR teammates during chest compressions.

The ultrasound examinations were conducted by two emergency medicine board-certified physicians, each of whom had previously performed POCUS-CAC at least ten times before study participation. One examiner had prior experience with POCUS-CAC through participation in our previous study [9]. The other examiner received specific training in the technique, consisting of a 1 h lecture and hands-on demonstration before study participation. Convenience sampling was employed during their shifts to select the study participants due to pandemic-related constraints and limited staff availability. A Samsung Ultrasound HM70A (Samsung Healthcare, Seoul, Korea) with a 7–16 MHz linear transducer with the vascular preset setting was used. Only B-mode imaging was used for evaluating POCUS-CAC.

### 2.4. Data Collection

The following data were collected: age, sex, past medical history (including hypertension, diabetes, and chronic renal or neurological disease), CPR-related information (the location of cardiac arrest, witness status, whether bystander CPR was administered, and use of mechanical CPR), no-flow duration (time from collapse to the start of CPR), and the rhythm detected using an automated external defibrillator (AED) by emergency medical services (EMS). Furthermore, the duration of CPR (pre-hospitalization and in the ED), initial ECG rhythms observed in the ED, and the probable causes of the cardiac arrest were recorded. Outcomes of the CPR efforts and the post-resuscitation ROSC status were also recorded. For each POCUS-CAC assessment, the POCUS-CAC finding and the circulatory status at the subsequent rhythm check were recorded. The CPR cycle and chronological order of POCUS-CAC assessments within each cycle were also recorded. When the reference-standard circulatory status at the subsequent rhythm check was unavailable, corresponding POCUS-CAC assessment was excluded from the primary diagnostic analysis. Missing data regarding patient demographics were categorized as miscellaneous.

### 2.5. Outcomes

The primary outcome was the diagnostic performance of POCUS-CAC during ongoing chest compressions for identifying circulatory status at the subsequent rhythm check. The secondary outcome was the diagnostic performance of POCUS-CAC during ongoing chest compressions for identifying circulatory status at the end of resuscitation.

### 2.6. Statistical Analysis

Continuous variables were summarized as means with standard deviation (SD) or medians with interquartile range (IQR), whereas categorical variables were presented as numbers and percentages. The diagnostic performance of POCUS-CAC during ongoing chest compressions, including accuracy, sensitivity, specificity, positive predictive value (PPV), negative predictive value (NPV), positive likelihood ratio, and negative likelihood ratio, was quantified. Accuracy, sensitivity, specificity, PPV, and NPV were expressed as percentages. The corresponding 95% confidence intervals (CIs) were derived using the percentile method from 5000 patient-level cluster bootstrap replicates. Patients were resampled with replacement, and all POCUS-CAC assessments from each selected patient were retained within each bootstrap sample.

For exploratory longitudinal analyses, diagnostic performance was evaluated according to the chronological order of POCUS-CAC assessments within each CPR cycle, with 95% CIs estimated using the same patient-level cluster bootstrap method. Serial POCUS-CAC patterns were also characterized among CPR cycles with at least two assessments.

Statistical analysis was performed using SAS version 9.4 (SAS Institute, Cary, NC, USA). Patient-level cluster bootstrap analyses were performed using R version 4.3.3 (R Foundation for Statistical Computing, Vienna, Austria).

## 3. Results

### 3.1. Characteristics of the Study Participants

**Figure 2:**
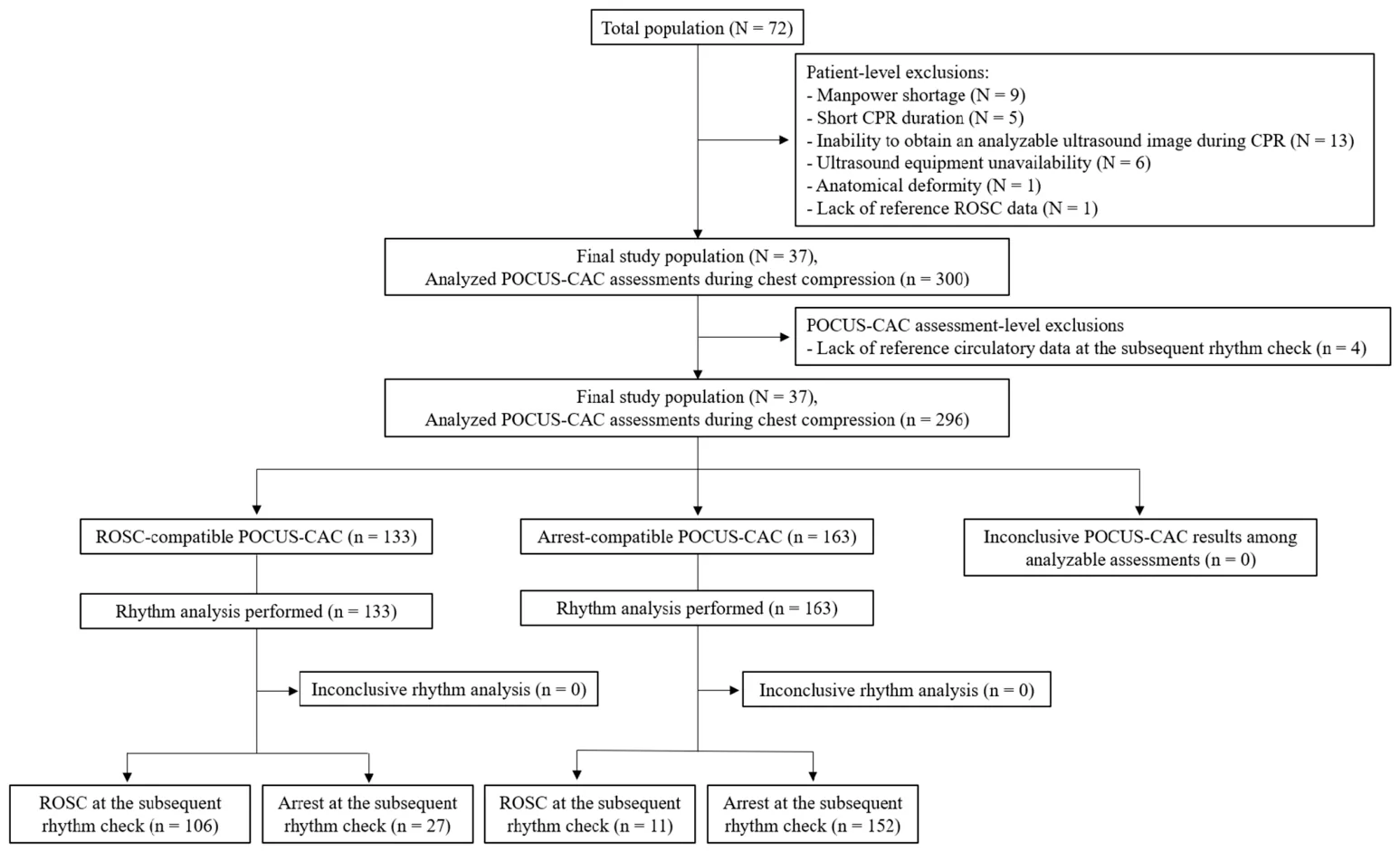
Study flow diagram. This study initially enrolled 72 patients, of whom finally 37 were included in the final analysis, yielding a total of 296 POCUS-CAC images for the primary analysis. Among these, 133 POCUC-CAC assessments were ROSC-compatible, and 163 images were arrest-compatible. Of the 133 ROSC-compatible assessments, 106 were confirmed as ROSC at the subsequent rhythm checks. Of the 163 arrest-compatible POCUS-CAC assessments, 152 were confirmed as arrest at the subsequent rhythm checks. *N* indicates the number of patients, and *n* indicates the number of POCUS-CAC assessments. Abbreviations: CPR, cardiopulmonary resuscitation; POCUS-CAC, point-of-care ultrasound-carotid artery compression; ROSC, return of spontaneous circulation.

A total of 72 patients were initially selected between June 2022 and October 2023. Among them, 33 were excluded owing to various challenges in the application of POCUS-CAC, such as manpower shortages (*N* = 9), brief duration of CPR (*N* = 5), inability to obtain analyzable ultrasound images (*N* = 13), and unavailability of ultrasound equipment (*N* = 6). One patient was excluded due to anatomical deformities of the neck, and another for lacking reference ROSC data at rhythm analysis. Consequently, 37 patients with 300 POCUS-CAC assessments were included in this study. Of these, 4 POCUS-CAC assessments were excluded from the primary diagnostic analysis due to lack of information regarding the subsequent rhythm analysis, leaving 296 POCUS-CAC assessments for analysis (Figure 2).

The mean age of the patients was 71 years (SD, 18), and 57% were male. Of the analyzed cohort, 73% (*N* = 27) experienced OHCA. In addition, 73% (*N* = 27) were witnessed, and bystander CPR was performed. All patients received mechanical CPR. The median no-flow time was 0 min (IQR, 0–7), and the median ED CPR time was 20 min (IQR, 9.5–23). The median number of POCUS-CAC assessments per patient was 6 (IQR 3–10; range 2–35). Among the 37 analyzed patients, 17 (46%) achieved ROSC as detailed in Table 1. No adverse events related to performing POCUS-CAC were reported.

### 3.2. Diagnostic Performance of POCUS-CAC During Ongoing Chest Compressions for Identifying Circulatory Status at the Subsequent Rhythm Check

POCUS-CAC during ongoing chest compressions accurately identified ROSC in 106 cases (true positives) and cardiac arrest in 152 cases (true negatives) at the subsequent rhythm check. However, ROSC-compatible findings were observed in 27 cases of cardiac arrest (false positives, FP), and arrest-compatible findings were observed in 11 cases of ROSC (false negatives, FN) (Table 2).

The sensitivity was 90.6% (95% CIs: 83.7–95.4), specificity was 84.9% (95% CIs: 74.5–92.6), and overall accuracy was 87.2% (95% CIs: 82.2–92.2). PPV was 79.7% (95% CIs: 70.6–87.3), and NPV was 93.3% (95% CIs: 87.3–97.3) (Table 3).

### 3.3. Diagnostic Performance of POCUS-CAC During Ongoing Chest Compressions for Identifying Circulatory Status at the End of Resuscitation

For the prespecified secondary outcome, POCUS-CAC showed a sensitivity of 59.3% (95% CI, 38.3–81.8), specificity of 59.4% (95% CI, 40.8–79.6), and overall accuracy of 59.3% (95% CI, 44.6–75.0) for identifying circulatory status at the end of resuscitation (Table 3). The corresponding cross-table is presented in Supplementary Table S1.

### 3.4. Exploratory Analyses of Serial POCUS-CAC Within CPR Cycle

In exploratory longitudinal analyses, diagnostic performance remained generally consistent across the first three POCUS-CAC assessment orders within CPR cycles. Sensitivity was 88.0% (95% CI, 80.0–95.1), 91.9% (95% CI, 81.8–100.0), and 90.0% (95% CI, 71.4–100.0), specificity was 83.2% (95% CI, 71.6–92.3), 86.0% (95% CI, 73.3–94.7), 87.0% (95% CI, 69.6–100.0), and accuracy was 84.8% (95% CI, 77.7–91.1), 88.3% (95% CI, 80.5–94.8), 88.4% (95% CI, 75.5–100.0) for the first, second, and third assessments, respectively (Supplementary Table S2). The fourth and fifth assessments were infrequent and were not included in the order-specific performance analyses.

Among CPR cycles with at least two POCUS-CAC assessments, all 45 cycles showing persistently arrest-compatible findings were followed by cardiac arrest at the subsequent rhythm check. Among 37 cycles showing persistently ROSC-compatible findings, 33 (89.2%) were followed by ROSC and 4 (10.8%) by cardiac arrest. Among 7 cycles showing an arrest-to-ROSC transition, 4 (57.1%) were followed by ROSC and 3 (42.9%) by cardiac arrest, whereas all 5 cycles showing ROSC-to-arrest transition were followed by cardiac arrest (Supplementary Table S3).

Patient-level serial POCUS-CAC trajectories according to CPR cycle and final circulatory status are shown in Supplementary Figure S1.

## 4. Discussion

In this prospective pilot diagnostic study, POCUS-CAC performed during ongoing mechanical chest compressions showed favorable diagnostic performance for identifying circulatory status at the subsequent rhythm check, with a sensitivity of 90.6% and an accuracy of 87.2%. Because POCUS-CAC allows emergency personnel to directly visualize carotid artery compressibility without interrupting chest compressions, it may provide a practical approach to circulatory assessment while minimizing hands-off time, consistent with 2025 American Heart Association (AHA) guidelines [13]. However, further validation across various emergency settings is required before its clinical implementation.

Diagnostic performance was lower when POCUS-CAC findings were compared with circulatory status at the end of resuscitation than when they were compared with circulatory status at the subsequent rhythm check. This difference likely reflects the distinct clinical meaning of the two reference outcomes. The primary analysis evaluated whether POCUS-CAC reflected circulatory status at the subsequent rhythm check within the same CPR cycle, whereas the secondary analysis compared individual POCUS-CAC findings obtained during ongoing CPR with the eventual circulatory status at the end of resuscitation. Because circulatory status may change during the course of resuscitation, an arrest-compatible or ROSC-compatible finding at a given assessment may accurately reflect the contemporaneous circulatory state but may not correspond to the eventual resuscitation outcome. Accordingly, the lower performance for the end-of-resuscitation outcome likely reflects the different temporal and clinical context of this secondary outcome rather than the ability of POCUS-CAC to reflect circulatory status at the time of assessment. These findings therefore support that POCUS-CAC may be more appropriately interpreted as a dynamic assessment of circulatory status during ongoing resuscitation rather than as a predictor of the final resuscitation outcome.

In the exploratory longitudinal analyses, the diagnostic performance of POCUS-CAC remained generally consistent across the first three assessments within the same CPR cycle. In addition, persistent POCUS-CAC patterns within the same CPR cycle were generally consistent with the circulatory status at the subsequent rhythm check. In contrast, transitional POCUS-CAC patterns showed mixed outcomes at the subsequent rhythm check. These findings suggest that serial assessment of POCUS-CAC may provide additional information regarding changes in circulatory status during ongoing CPR. However, as this analysis was exploratory, further studies are needed to confirm the clinical significance of these serial patterns.

The occurrence of FP and FN offers valuable insights into the limitations and potential advantages of POCUS-CAC during ongoing chest compressions. Among the 15 patients with 27 FP outcomes, 10 cases were observed in the transition between ROSC and arrest, suggesting that a weak pulse ROSC may have been present. However, it might be missed by manual pulse detection, which served as the reference for ROSC determination. This finding aligns with previous studies that have proposed the concept of pseudo-pulseless electrical activity [14,15,16,17]. Given that manual detection is less reliable for weak pulses [18], direct visualization using POCUS-CAC may be more sensitive in such cases. Moreover, in the 11 FN cases, excessive probe pressure beyond necessary for vein compression due to potentially incorrect visual determination of complete internal jugular vein collapse may have caused artificial compression of the carotid artery, leading to false arrest predictions. Standardizing probe pressure and further investigating the relationship between carotid artery compressibility and blood pressure will help refine POCUS-CAC techniques and improve its accuracy.

The POCUS-CAC during ongoing chest compressions identified the shift from arrest-compatible to ROSC-compatible findings in eight patients. Serial POCUS-CAC assessments occasionally demonstrated that the carotid artery became progressively less compressible across multiple CPR cycles, and in some cases within the same cycle (Supplementary Video S2). However, owing to the binary classification of compressibility into either compressible or non-compressible in this study, these gradual changes were not quantitatively analyzed. Recent studies have demonstrated the feasibility of frame-by-frame quantification of carotid artery compressibility using artificial intelligence, as well as an inverse relationship between quantitatively measured carotid artery compressibility and arterial blood pressure [19,20]. The observed relationship between carotid artery compressibility and arterial blood pressure suggests that carotid compressibility may reflect a continuum of underlying hemodynamic status rather than a purely binary phenomenon. Future application of quantitative approaches to serial POCUS-CAC assessment during CPR may allow more detailed characterization of changes in circulatory status.

Among cases predicted as cardiac arrest, the carotid artery was compressed during diastolic phase, and it was fully compressed even during systolic phase in some cases (Supplementary Video S3A). As chest compressions generate 20–50% of normal cardiac output [21,22] during the systolic phase, systemic flow from the chest compression should have maintained some arterial patency (Supplementary Video S3B). This observation creates uncertainty on the quality of CPR being delivered, such as the position or depth of chest compressions. In light of this, whether carotid artery compressibility during chest compressions is associated with CPR quality or hemodynamic effectiveness was not evaluated in the present study and warrants further investigation.

It is notable that 10 among 37 patients consistently showed arrest on POCUS-CAC during ongoing chest compressions throughout the resuscitation process, and 9 (90%) ultimately died. Although this finding should be interpreted cautiously given the small sample size, persistent arrest-compatible POCUS-CAC may represent a refractory cardiac arrest state despite ongoing CPR. As patients with refractory cardiac arrest may be candidates for advanced rescue strategies such as extracorporeal CPR, whether persistent arrest-compatible POCUS-CAC could contribute to the early identification of such patients warrants prospective investigation.

Physiological parameters such as end-tidal carbon dioxide (ETCO_2_) and diastolic arterial pressure are commonly used to assess CPR quality and predict ROSC; however, both have limitations. ETCO_2_ is widely used in clinical settings owing to its ease of use and non-invasive nature. However, ETCO_2_ readings can be affected by various external factors, including the administration of drugs such as sodium bicarbonate, epinephrine, or excessive ventilation during resuscitation efforts. These factors can alter carbon dioxide levels independently of circulation status, reducing the reliability of ETCO_2_ as a sole predictor of ROSC [23]. Diastolic arterial pressure, though a more direct measure of hemodynamic effects, requires invasive techniques that may not be feasible in emergency settings [24].

Since the use of carotid Doppler ultrasound in a CPR setting in 2015, it has been explored in the fields of resuscitation, for example, as an alternative to manual pulse detection and a tool for assessing CPR efficacy during cardiac arrest [25,26,27,28]. Although many studies have been conducted to compare the efficacy between Doppler ultrasound and capnography in a CPR setting, its reliability for evaluating CPR performance is controversial [29,30]. Additionally, detecting pulse during cardiac arrest with femoral Doppler ultrasound has a greater accuracy than manual pulse palpation, with a peak systolic velocity cutoff of 20 cm/s effectively identifying systolic blood pressures above 60 mmHg [31]. While these efforts are promising, achieving accurate Doppler readings requires stable alignment with the vessel, which can be challenging to maintain during ongoing chest compression. Furthermore, Doppler studies demand specialized expertise and are prone to artifacts caused by chest compressions, limiting their real-time use. In contrast, POCUS-CAC is simpler to implement in emergency settings and avoids the complexities and potential delays associated with other pulse determination modalities [26].

This study has several limitations. First, the study population was limited, as the sample size was not predetermined due to the lack of prior studies, and the single-institution setting further restricts the generalizability of the findings. Additionally, convenience sampling may have affected sample diversity. Second, carotid artery compressibility was assessed visually using predefined B-mode criteria, without quantitative assessment. Although visual assessment may facilitate rapid evaluation during CPR, it may introduce operator-dependent variability. Third, clinical determination of ROSC remains challenging, particularly because manual pulse palpation has important limitations [14,15,24]. We relied on a clinical decision based on manual pulse palpation and the patient’s ECG rhythm according to the 2020 AHA guidelines [3]. However, this method may not be reliable, and future studies should incorporate emerging standards for more accurate ROSC detection. Fourth, the application and interpretation of POCUS-CAC during ongoing chest compressions were challenging owing to the dynamic nature of chest compressions. Motion artifacts added complexity to interpretation. This issue may limit its reproducibility by less experienced clinicians. The unpredictable nature of CPR occasionally prevented POCUS-CAC assessments from being performed at uniform intervals, potentially impacting data collection consistency. In addition, analyzable POCUS-CAC images could not be obtained in 13 patients during CPR, which may limit the real-world feasibility of this technique. Fifth, carotid artery disease may affect carotid artery compressibility, but in the real CPR environment, it was difficult to identify patients’ medical history prior to POCUS-CAC application. Lastly, despite efforts made to blind physicians interpreting POCUS-CAC results, complete blinding may not have been maintained in the dynamic CPR setting, potentially introducing bias in the interpretation of the results.

In this prospective pilot diagnostic study, POCUS-CAC during mechanical CPR showed favorable diagnostic performance for identifying circulatory status at subsequent rhythm checks. Serial POCUS-CAC assessments may provide additional information regarding changes in circulatory status during ongoing CPR, while persistent arrest-compatible findings warrant further investigation as a potential marker of refractory cardiac arrest. Further research with larger, multicenter studies is needed to validate these findings and determine the clinical role of POCUS-CAC in resuscitation.

## Figures and Tables

**Table 1:** Basic characteristics of study participants.

Characteristics	Patients (*n* = 37)	Characteristics	Patients (*n* = 37)
Age, year (mean, SD)	71 (18)	Initial ED ECG rhythm	
Male (%)	21 (57)	Asystole	22 (60)
Past medical history		PEA	11 (30)
HTN	12 (32)	Ventricular Tachycardia	3 (8)
DM	7 (19)	Ventricular Fibrillation	1 (3)
Dyslipidemia	4 (11)	No-flow time, min (median, IQR)	0 (0–7)
Chronic heart disease	8 (22)	EMS CPR time, min (median, IQR)	20 (10–22.5)
Chronic lung disease	1 (3)	ED CPR time, min (median, IQR)	20 (9.5–23)
Chronic liver disease	2 (5)	Cause of arrest	
Chronic renal disease	2 (5)	Cardiogenic	4 (11)
Chronic neurological disease	5 (14)	Hypoxia	4 (11)
Malignancy	11 (30)	Metabolic	11 (30)
Location of cardiac arrest		Trauma	2 (5)
OHCA	27 (73)	Unknown	16 (43)
Home	15 (41)	Reason to stop CPR	
Street	3 (8)	ROSC	17 (46)
Work	4 (11)	DNR	1 (3)
Others	5 (13)	No possibility of ROSC	19 (51)
IHCA	10 (27)	ECMO	0 (0)
Witnessed arrest	27 (73)	Prognosis after CPR	
Bystander CPR	27 (73)	No ROSC	20 (54)
Mechanical CPR	37 (100)	ROSC	17 (46)
EMS AED rhythm		Death within 24 h	10 (27)
Unknown/Miscellaneous	12 (32)	Death after 24 h	5 (14)
Non-shockable	21 (57)	Survival discharge	2 (5)
Shockable	4 (11)	POCUS-CAC assessments per patient (Median, IQR), Range	6 (3–10), 2–35

Data are presented as numbers (percentage, %), mean (SD), and median (IQR). Abbreviations: AED, automated external defibrillator; CPR, cardiopulmonary resuscitation; DM, diabetes mellitus; DNR, do not resuscitate; ECG, electrocardiogram; ECMO, extracorporeal membrane oxygenation; ED, emergency department; EMS, emergency medical services; HTN, hypertension; IHCA, in-hospital cardiac arrest; IQR, interquartile range; OHCA, out-of-hospital cardiac arrest; PEA, pulseless electrical activity; POCUS-CAC, point-of-care ultrasound-carotid artery compression; ROSC, return of spontaneous circulation; SD, standard deviation.

**Table 2:** Cross-table of POCUS-CAC findings and circulatory status at the subsequent rhythm check (*n* = 296).

			Rhythm Analysis		
		ROSC	Arrest	Others	Total
POCUS-CAC during Ongoing Chest Compressions	ROSC	106	27	0	133
	Arrest	11	152	0	163
	Others	0	0	0	
	Total	117	179		296

Shaded cells indicate concordant classifications between POCUS-CAC findings and circulatory status at the subsequent rhythm check. The ‘Others’ category comprises cases with missing data or inconclusive test results. Abbreviations: CPR, cardiopulmonary resuscitation; POCUS-CAC, point-of-care ultrasound-carotid artery compression; ROSC, return of spontaneous circulation.

**Table 3:** Diagnostic performance of POCUS-CAC during ongoing chest compressions for identifying circulatory status at the subsequent rhythm check and at the end of resuscitation.

Diagnostic Measure	Subsequent Rhythm Check (*n* = 296) (95% CI)	End of Resuscitation (*n* = 300) (95% CI)
Sensitivity, %	90.6 (83.7–95.4)	59.3 (38.3–81.8)
Specificity, %	84.9 (74.5–92.6)	59.4 (40.8–79.6)
PPV, %	79.7 (70.6–87.3)	35.0 (15.7–63.9)
NPV, %	93.3 (87.3–97.3)	79.8 (60.2–93.6)
Accuracy, %	87.2 (82.2–92.2)	59.3 (44.6–75.0)
Positive likelihood ratio	6.01 (3.56–12.01)	1.45 (0.82–3.14)
Negative likelihood ratio	0.11 (0.05–0.19)	0.69 (0.29–1.21)

95% confidence intervals were estimated using 5000 patient-level cluster bootstrap replicates. Four POCUS-CAC assessments were excluded from the subsequent rhythm check analysis because the corresponding reference standard circulatory status was unavailable. Abbreviations: CPR, cardiopulmonary resuscitation; NPV, negative predictive value; POCUS-CAC, point-of-care ultrasound-carotid artery compression; PPV, positive predictive value; ROSC, return of spontaneous circulation.

## Data Availability

Data related to this study cannot be released due to the information security policies of the hospitals.

## Supplementary Materials

The following supporting information can be downloaded at: https://www.mdpi.com/article/10.3390/diagnostics16182956/s1, Table S1. Cross-table of POCUS-CAC findings and circulatory status at the end of resuscitation (*n* = 300). Table S2. Exploratory diagnostic performance of POCUS-CAC according to assessment order within CPR cycles. Table S3. Serial POCUS-CAC patterns within CPR cycles with at least two assessments. Figure S1. Patient-level trajectories of serial POCUS-CAC assessments during CPR. Each row represents an individual patient. POCUS-CAC assessments are displayed according to CPR cycle and chronological order within each cycle. Symbols indicate the circulatory status determined at the subsequent rhythm check. Patients are grouped according to circulatory status at the end of resuscitation. The horizontal axis represents CPR cycle rather than exact elapsed CPR time. Abbreviations: CPR, cardiopulmonary resuscitation; POCUS-CAC, point-of-care ultrasound-carotid artery compression; ROSC, return of spontaneous circulation. Video legends: Video S1. POCUS-CAC During Chest Compressions. A: ROSC-compatible; B: arrest-compatible. If the carotid artery remains uncompressed while the internal jugular vein is fully compressed during both diastole and systole, the POCUS-CAC assessment is classified as ROSC-compatible. If the carotid artery is fully compressed while the internal jugular vein is fully compressed during the diastole period, the POCUS-CAC assessment is classified as arrest-compatible. Abbreviations: POCUS-CAC, point-of-care ultrasound-carotid artery compression; ROSC, return of spontaneous circulation. Video S2. Serial Videos of POCUS-CAC During Ongoing Chest Compressions of the 30th Enrolled Patient. An example of the carotid artery becoming progressively less compressible was observed over multiple CPR cycles, eventually leading to ROSC. A: in the 1st cycle; B: in the 2nd cycle; C: in the 3rd cycle (first 30 s); D: in the 3rd cycle (1 min). Abbreviations: CPR, cardiopulmonary resuscitation; POCUS-CAC, point-of-care ultrasound-carotid artery compression; ROSC, return of spontaneous circulation. Video S3. Degree of Carotid Artery Collapse During POCUS-CAC in the Systolic Phase of Chest Compressions. A: complete collapse; B: incomplete collapse. An example of a fully compressed carotid artery during systolic phase of Intra-CPR POCUS-CAC, which may be indicative of poor CPR quality. An example of an incompletely compressed carotid artery during systolic phase of Intra-CPR POCUS-CAC, which may indicate fair CPR quality. Abbreviations: CPR, cardiopulmonary resuscitation; POCUS-CAC, point-of-care ultrasound-carotid artery compression.

## Abbreviations

POCUS, point-of-care ultrasound; POCUS-CAC, POCUS-carotid artery compression; ROSC, return of spontaneous circulation; CPR, cardiopulmonary resuscitation; ED, emergency department; OHCA, out-of-hospital cardiac arrest; IHCA, in-hospital cardiac arrest; ACLS, advanced cardiovascular life support; ECG, electrocardiogram; AED, automated external defibrillator; EMS, emergency medical services; SD, standard deviation; IQR, interquartile range; PPV, positive predictive value; NPV, negative predictive value; CIs, confidence intervals; AHA, American Heart Association; FP, false positive; FN, false negative; ETCO_2,_ end-tidal carbon dioxide.
