# Supplementary material for: Serial Ultrasound-Guided Carotid Artery Compression During Chest Compressions for Assessing Circulatory Status: A Prospective Pilot Diagnostic Study
Source: Diagnostics (Basel). 2026 Sep 13;16(18):2956. doi: 10.3390/diagnostics16182956 (PMC13605083; doi:10.3390/diagnostics16182956)
Supplement: Supplementary file 1 [file diagnostics-16-02956-s001.zip › Supplementary_table_260906.pdf]

Supplementary Table S1. Cross-table of POCUS-CAC findings and circulatory status at the end of resuscitation (n = 300)

		Circulatory Status at the End of Resuscitation			
		ROSC	Arrest	Others	Total
POCUS-CAC during Ongoing Chest Compressions	ROSC	48	89	0	137
	Arrest	33	130	0	163
	Others	0	0		
	Total	81	219		300

The 'Others' category comprises cases with missing data or inconclusive test results. Abbreviations. CPR, cardiopulmonary resuscitation; POCUS-CAC, point-of-care ultrasound-carotid artery; ROSC, return of spontaneous circulation.

Supplementary Table S2. Exploratory diagnostic performance of POCUS-CAC according to assessment order within CPR cycles

Assessment order	Number of assessments	Sensitivity (95% CI)	Specificity (95% CI)	Accuracy (95% CI)
1	145	88.0 (80.0–95.1)	83.2 (71.6–92.3)	84.8 (77.7–91.1)
2	94	91.9 (81.8–100.0)	86.0 (73.3–94.7)	88.3 (80.5–94.8)
3	43	90.0 (71.4–100.0)	87.0 (69.6–100.0)	88.4 (75.5–100.0)
4	12	NA	NA	NA
5	2	NA	NA	NA

The number of assessments within each CPR cycle varied according to clinical circumstances, resulting in a small number of fourth and fifth assessments. Diagnostic performance measures for the fourth and fifth assessment orders were not reported because of the small number of observations. Confidence intervals for the first three assessment orders were estimated using 5,000 patient-level cluster bootstrap replicates. Abbreviations. NA, not applicable; CI, confidence interval.

Supplementary Table S3. Serial POCUS-CAC patterns within CPR cycles with at least two assessments

Serial POCUS-CAC pattern	Number of CPR cycles	Cardiac arrest at subsequent rhythm check	ROSC at subsequent rhythm check
Persistent arrest-compatible	45	45 (100.0%)	0 (0.0%)
Persistent ROSC-compatible	37	4 (10.8%)	33 (89.2%)
Arrest-to-ROSC transition	7	3 (42.9%)	4 (57.1%)
ROSC-to-arrest transition	5	5 (100.0%)	0 (0.0%)
Total	94	57 (60.6%)	37 (39.4%)

The serial patterns analysis was restricted to CPR cycles with at least two POCUS-CAC assessments. Abbreviations. CPR, cardiopulmonary resuscitation; POCUS-CAC, point-of-care ultrasound-carotid artery; ROSC, return of spontaneous circulation.
